# Future-Proofing European Pharmaceutical Regulatory and Market Access Practices Based on EU Learnings from the COVID-19 Pandemic: Insights from Multi-Stakeholder Interviews

**DOI:** 10.1007/s43441-025-00855-2

**Published:** 2025-09-06

**Authors:** Zilke Claessens, Grace Beirne, Catherine Decouttere, Nico Vandaele, Liese Barbier, Isabelle Huys

**Affiliations:** 1 Clinical Pharmacology and Pharmacotherapy, Department of Pharmaceutical and Pharmacological Sciences, KU Leuven, Leuven, Belgium; 2 Access-To-Medicines Research Centre, Faculty of Economics & Business, KU Leuven, Leuven, Belgium

**Keywords:** COVID-19, Regulatory framework, Market access, Patient access, Joint procurement, Pandemic preparedness, Qualitative research

## Abstract

**Introduction:**

During the COVID-19 pandemic, regulatory and market access actions were taken to expedite the market entry of COVID-19 medicines. This study aims to (i) capture multi-stakeholder views on these actions, and (ii) provide recommendations for future-proofing routine and health-emergency frameworks.

**Methods:**

Semi-structured interviews were conducted with policy makers/advisors (i.e. regulators, HTA assessors, and payers), and pharmaceutical industry representatives across Europe to elicit their perspectives on marketing authorisation and market access practices during the COVID-19 pandemic. Interviews were transcribed ad verbatim and transcripts analysed via the thematic framework method.

**Results:**

The interviews (*n* = 16) resulted in an overview of stakeholder-perceived benefits and limitations for four key regulatory advice or authorisation procedures (i.e. emergency task force, rapid scientific advice, rolling review, conditional marketing authorisation) and one market access procedure (i.e. joint procurement) applied during the COVID-19 pandemic. Highlighted benefits of the procedures relate to a reduction in timelines, enhanced collaboration and alignment, procedural flexibilities, and often a combination of these. Challenges are linked to inefficient allocation of time and resources for both industry representatives and policymakers/advisors and decreased transparency in certain procedures. In addition, several recommendations for the optimisation of both the routine and health-emergency healthcare framework were proposed. Emphasis is placed on the need for enhanced interaction and alignment between industry representatives and policymakers/advisors but also within stakeholder groups, development of more pragmatic and flexible procedures, and application of clear and transparent eligibility criteria for facilitating actions.

**Conclusion:**

This study provides an overview of the perceptions from regulatory, and market access practices during COVID-19, highlighting how these experiences can inform regulatory and market access practices both in routine times and during health emergencies. Taking stock of stakeholder reflections and lessons learned are valuable for improving preparedness and responsiveness in future health crises.

**Supplementary Information:**

The online version contains supplementary material available at 10.1007/s43441-025-00855-2.

## Introduction

COVID-19 brought many unique challenges and expectations for society in terms of pharmaceutical development and evaluation. There was high demand for new pharmaceutical innovations to reach the market rapidly, which called for a huge acceleration of marketing authorization and market access processes. To meet this need, the European Commission launched an European Union (EU) strategy for COVID-19 vaccines in June 2020 and in 2021 also one for COVID-19 therapeutics [1, 2]. Following these efforts of policy makers and developers, there was a large increase in research activities in this field. For example, 71 clinical trial data packages for COVID-19 vaccines and therapeutics were published during the public health emergency [3]. Development and authorization timelines were greatly reduced, particularly for vaccines [4]. While mRNA vaccine platforms had been under development for over a decade, the clinical development and regulatory approval phases during COVID-19 were significantly accelerated, with timelines reduced from the usual 10–15 years to approximately 1–2 years for these stages alone [5]. Additionally, there was an increase in the number of antivirals and vaccines in the development pipeline overall [4]. The first (repurposed) antiviral was authorised within four months after the declared start of the pandemic and the first vaccine after nine months [6, 7].

The global impact of the COVID-19 pandemic together with governmental actions were drivers that increased and accelerated COVID-19 R&D [8]. These rapid developments were also able to reach the market faster due to accelerated regulatory assessment speed, mainly due to a rolling review process which was introduced by the European Medicines Agency (EMA) [4, 9]. Unlike the standard process, where all data on quality, safety, and efficacy must be consolidated and submitted as a single, complete dossier before evaluation begins, the rolling review allows companies to submit data packages and study results to the EMA as soon as they become available from ongoing studies [10]. This procedure significantly reduced the time to marketing authorisation and was pivotal in accelerating access to safe and effective products during the pandemic [10]. Moreover, the Emergency Task Force (ETF), a multidisciplinary group of regulatory and clinical experts, established under Regulatory (EU) 2022/123, was mobilized to provide scientific advice, support clinical trial coordination, and provide recommendations for the use of emergency medicinal products [11].

In terms of market access, before COVID-19, the evaluation process would generally begin only after a national dossier was submitted following centralized marketing authorization. During the pandemic, the health technology assessment (HTA) and negotiations already started before the European marketing authorization was granted, so that medicines could become available almost instantly at the time of European marketing authorization [12]. Additionally, National Immunization Technical Advisory Groups (NITAGs) played a crucial role by providing independent, evidence-based recommendations on vaccine use and prioritization at the national level, complementing the work of HTA bodies. For procurement and stockpiling, the European Commission coordinated a joint procurement process, allowing EU member states to collectively negotiate and purchase vaccines and other medical countermeasures.

The COVID-19 pandemic necessitated an unprecedented acceleration of the regulatory and market access frameworks to ensure that crucial medical products were rapidly made available to citizens across European member states. The current post-pandemic period offers a valuable opportunity to reflect on these practices, identify their strengths and weaknesses, and derive lessons to enhance future regulatory and market access frameworks. Therefore, this study aims to capture and compare the perspectives of developers and policymakers/advisors regarding the regulatory and market access practices employed during the pandemic. By analysing these insights, the study seeks to identify ways to strengthen the existing regulatory and market access tools for innovative medicines and improve preparedness for future health emergencies.

## Methods

This study made use of qualitative methods, more specifically semi-structured interviews with both industry stakeholders and policymakers/advisors. Participants were sampled across European member states and organizations to ensure diverse perspectives on regulatory and market access practices during the COVID-19 pandemic. Ethical clearance for the study was obtained from Ethics Committee Research UZ / KU Leuven (S66267).

### Participant Selection and Recruitment

Semi-structured interviews were conducted with experts from two stakeholder groups: pharmaceutical industry and policymakers/advisors, to obtain multi-stakeholder insights on regulatory and market access practices applied during the COVID-19 pandemic in the European context. Participants were purposively sampled based on specific suitability criteria [13], including: (i) professional involvement with regulatory processes or market access activities during the COVID-19 pandemic, (ii) geographic scope of responsibility (national, European, or global), and (iii) representation across different types of organizations (pharmaceutical companies actively involved in vaccine or therapeutic development, trade associations, regulatory bodies, HTA bodies, and policy institutions). Participants from pharmaceutical companies were selected specifically based on their active involvement in COVID-19-related products (vaccines and/or therapeutics) to ensure relevant insights.

Potential participants were contacted via email to invite them for individual interviews. Upon request of the invited participant, it was possible to invite a colleague from the same organization. Snowball sampling was utilized to identify additional participants through referrals from initial participants. Participants were recruited until data saturation was reached using the code frequency count method with a stopping criterion of no new codes for three consecutive interviews (see Supplementary material file 1) [14].

### Conduct of the Interviews

A semi-structured interview guide was developed based on a review of existing literature, and piloted with one expert for each stakeholder group (Supplementary material file 2). The guide included open-ended questions aimed at exploring stakeholders’ perspectives on measures utilized during the COVID-19 pandemic regarding vaccine and therapeutic regulatory and market access procedures. The topic guide was limited to exploring measures and challenges related to scientific advice/regulatory assessment, and market access procedures. The interviews were performed between May 2022 and November 2022. Interviews were conducted via Microsoft Teams to facilitate participation, held in English, and took on average 51 min, allowing for in-depth exploration of participants’ insights and experiences.

### Analysis

The online interviews were digitally audio-recorded, transcribed ad verbatim, and subsequently pseudonymized to ensure participant confidentiality. The thematic framework method was employed for data analysis using Nvivo software^®^ [15]. A thematic framework extraction table was developed based on literature and transcript reading by one researcher and discussed and agreed upon among co-authors. The coding was performed in Nvivo by one researcher and validated by a second researcher, with additional codes added as necessary. Data was extracted from Nvivo and summarized in Excel, including both individual stakeholder statements and aggregated results per code. The in-depth analysis, conceptualisation, and synthesis was collaboratively performed by the two researchers and interpretations were discussed with the whole research team. This method allowed for systematic exploration of themes and patterns within the data.

This study focused on five key regulatory and market access measures (i.e. ETF, rapid scientific advice, rolling review, conditional marketing authorisation (CMA), and joint procurement) because these were the elements most frequently identified by participants (*n* > 3) as central to the COVID-19 response. Rather than selecting measures a priori, participants were asked to reflect on the procedures they found most relevant or impactful during the pandemic. This approach allowed for an open exploration of stakeholder perspectives. While other flexibilities (Supplementary material file 3) were occasionally mentioned, this manuscript concentrates on the measures most commonly discussed in depth across interviews.

## Results

There were 16 interviews performed (Table 1), with a total of 18 participants having active experience and/or knowledge on COVID-19 regulatory and/or market access practices.

**Table 1:** Participant characteristics. EU: European union, MA: market access, NA: not applicable, RA: regulatory affairs

Stakeholder group	Organisation type	Expertise		Geographical scope			Total Participants
		RA	MA	National	EU	Global	
Industry	Pharmaceutical company	3	2	2	3	0	5
	Pharmaceutical umbrella trade organisation	3	2	1	4	0	5
Policymaker/ advisor	Health Technology Assessment body	NA	NA	1	1	1	3
	Policy institution	NA	NA	0	1	1	2
	Marketing authorisation authority	NA	NA	2	1	0	3
Total		6	4	6	10	2	18

Note. There were 16 interviews conducted with a total of 18 participants (above table shows numbers at the participant level), two interviews with industry consisted of two participants each

**Fig. 1:**
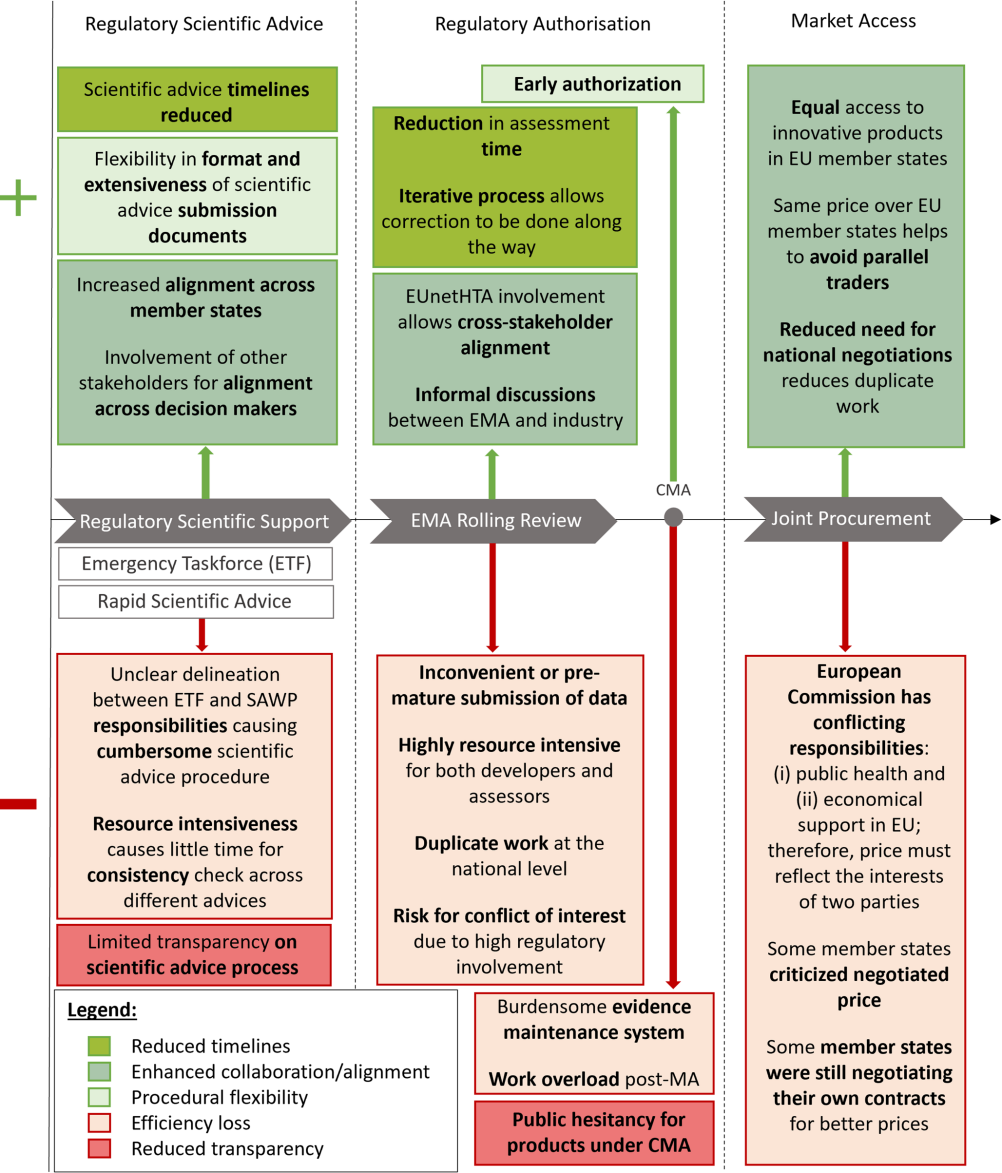
Overview of key benefits and limitations of pandemic regulatory and market access measures during the COVID-19 crisis according to interviewed stakeholders. CMA: Conditional Marketing Authorisation, EU: European Union, EMA: European Medicines Agency, ETF: Emergency Task Force, MA: Marketing Authorisation, RWE: Real World Evidence, SAWP: Scientific Advice Working Party

Based on the interviews, the EMA ETF, rapid scientific advice, rolling review, CMA, and joint procurement were identified by participants as key regulatory / market access measures during the pandemic. Figure 1 provides an overview of most mentioned benefits and limitations of these regulatory / market access measures. The benefits brought forward by participants can be grouped into three main categories: (i) reduced timelines, (ii) enhanced collaboration, and (iii) procedural flexibilities. On the other hand, there are some important unintended consequences, associated with these practices. These can be grouped into two categories: (i) efficiency loss and (ii) reduced transparency. Detailed explanations of benefits and limitations of each item follow below.

### Regulatory Scientific Support

#### Reported Strengths and Limitations of Pandemic Practices

Scientific advice was criticized by industry participants and policymakers/advisors as typically lengthy, rigid, and inefficient. However, during the COVID-19 pandemic, regular timelines for scientific advice were shortened, resulting in rapid scientific advice. These reduced timelines were achieved through efforts by the EMA and national regulatory authorities. Industry participants noted more efficient engagement in the scientific advice procedure, facilitated by proactive and iterative interactions between developers and assessors. Some scientific advice was reported to be issued outside the official procedure.

Industry participants reported greater acceptance of real-world evidence in regulatory and market access decision-making during the pandemic, alleviating pressure on lengthy clinical trials. Clinical trial flexibilities, such as remote participation, multiple laboratories for data assessment, and platform trials, were believed by industry participants to increase efficiency. Both stakeholder groups reported that greater flexibility in regulatory submission formats expedited processes, eliminating delays from changing or amending submission formats. These changes improved information transfer and expedited the process overall.

Duplication of efforts was reported as a significant source of inefficiency, with scientific advice provided at both European (i.e. EMA) and national levels. One policymaker/advisor explained that some companies preferred to first get scientific advice at the national level in member states with specialised expertise before seeking advice from the EMA. The ETF was crucial in providing scientific advice and coordinating regulatory activities, but duplication of efforts between the ETF and the Scientific Advice Working Party (SAWP) caused confusion in the delineation of responsibilities. Industry representatives mentioned that applicants were often unaware of internal deliberations and which expert groups were involved in assessment processes. Participants believed that early assignment of responsibilities could have reduced this duplication. One assessor reported inconsistencies in the structure and, in some cases, the content of scientific advice provided by EMA across different products, noting that advice was not always systematically aligned, partly due to limited time for cross-checking during the pandemic.

#### Optimisation Avenues

Rapid scientific advice was deemed by industry representatives to be a useful tool for early dialogue and hope this tool could be used to accelerate the development of products that address “unmet medical needs” or for “life-saving” products. Policymaker/advisors, however, think the procedure is too resource-intensive to be applied in routine practice. Nevertheless, they believe that a more pragmatic application procedure could lead to flexibilities that facilitate and accelerate submissions. Overall, policymakers/advisors agree that rapid scientific advice has low application potential, due to the time intensity.

*“I think if there are products which really are life-saving for patients*,* it would certainly be valuable to shorten the timeline. Because otherwise*,* your normal scientific advice timeline is between 40 to 70 days.”* (IND6).

“*Scientific advice is usually quite fast*,* so you can have your scientific advice in 30 days if necessary. Further acceleration is probably not really meaningful*,* especially when you consider the time that applicants usually take to prepare for scientific advice.”* (PMA6).

While industry representatives unanimously supported rapid scientific advice for future health emergencies, policymakers/advisors had varied responses. Increased support and capacity at national regulatory authorities were recommended to make this process feasible. The ETF was widely considered successful for EMA scientific advice during health emergencies. To avoid duplication with the SAWP, participants recommended clearly outlining the ETF’s responsibilities. One participant suggested the ETF should identify promising new products early in a pandemic and proactively circulate new information to national authorities, the EC, and the EMA to support institutional preparedness and continuity in decision-making across the network. Both stakeholder groups agreed on the high potential usefulness of the ETF going forward.

### EMA Rolling Review

#### Reported Strengths and Limitations of Pandemic Practices

The rolling review process allowed developers to receive ongoing feedback on their submissions, addressing potential delays early. Participants reported reduced regulatory timelines due to parallel processing of steps. Industry participants noted close collaboration with the EMA, with continuous, iterative advice on COVID-19 vaccine and therapeutic development. The EMA’s OPEN initiative also involved other non-EU regulatory authorities, enhancing transparency and sharing expertise.

“*EMA provided continuous*,* iterative advice on the development of the COVID vaccines and therapeutics. In particular*,* we had a constant dialogue with the regulators when we were developing the vaccine. Sort of like largely sitting alongside us as we’re seeing*,* analysing*,* and making decisions based upon the data from the trial*.” (IND1).

However, many participants cautioned that the rapid review timelines placed pressure on regulatory authorities, forcing them to halt other activities to focus on COVID-19-related dossiers. This was believed by both industry participants and policymakers/advisors to be challenging given their limited resources and the dual roles many committee members played as both EU assessors and national positions. Participants felt they were resource-intensive for both assessors and applicants and sometimes inefficient. Regulators reported that the rolling review increased personnel commitments and total time spent on reviewing submission dossiers and questioned whether resources were deployed most efficiently, despite faster outcomes.

*“The disadvantages are that you might prematurely submit something and then regulators spend a lot of their time looking at it and then that’s a waste of time because it turns out that the product is not working or the safety profile isn’t good enough. So you can waste your assessor’s time and assessors time is very precious.”* (IND8).

Moreover, several participants noted that some member states conducted additional national regulatory evaluations, leading to duplicated efforts and unequal access to treatments across Europe. One industry participant raised concern that the close and continuous involvement of regulators in data review during the pandemic risked conflict of interest by blurring the line between evaluation and co-development, potentially affecting perceived objectivity. Additionally, industry participants highlighted that EMA processes were still slower compared to the FDA in the United States, suggesting potential for further acceleration.

All participants agreed that procedural flexibilities, such as reliance on digital support (e.g. digital informed consent, electronic product information, remote meetings), approval of decentralised trials, and early results-based approvals, facilitated and expedited clinical evidence generation during the pandemic. However, several participants reported that the EU Clinical Trials Regulation (CTR) 536/2014 was not yet fully implemented during the early phase of the pandemic. This lack of full implementation was perceived by participants to have contributed to a fragmented clinical trial landscape, with numerous small, underpowered trials insufficient for drawing meaningful conclusions. While the CTR and the Clinical Trials Information System (CTIS) do not prevent the conduct of small trials, participants believe that the absence of a harmonized, centralized process made it challenging for regulators to prioritize and coordinate resources. This proliferation of small trials was believed to create an overload for clinical trial assessors and limited the generation of robust, generalizable evidence. Participants advocated for larger, multi-country trials for robust data and reliable outcomes. The rolling review process allowed regular evidence submission, several policymakers/ advisors noted that the submitted data was often immature, leading to inefficiencies in both data generation and assessment.

#### Optimisation Avenues

Overall, both stakeholder groups agreed that the rolling review should be implemented in a future health emergency but there was no consensus on its application in routine practice. Most policymakers/advisors found it too resource-intensive, favouring the PRIME scheme and accelerated assessment for routine use. Industry representatives did not rule out its case-specific application, citing insufficient tools for accelerating authorisation. Both groups agreed that more resources for regulators and clear requirements are essential for applying the rolling review in both routine practice and health emergencies. In routine practice, some suggested limiting its use to products addressing unmet medical needs, innovations, or public health interests. The simplified reporting format from the rolling review was seen as valuable for enhancing routine submission processes.

*“There are lessons learned that can be taken to see whether it is possible to work for the assessment of a medicine in a more iterative way. Not the rolling review*,* but in a more iterative way that you’re providing information when you think that you have a sufficient package of information.”* (IND2).

Both policymakers/advisors and industry representatives expressed support for an EU clinical trial approval system, with one protocol for a multinational study, promoting harmonisation across the EU and resource efficiency (endorsed under Regulation (EU) No 536/2014). Industry representatives additionally underlined that there is a need for more aligned evidence requirements for submission between the different member states but also between stakeholders (e.g. HTA bodies, payers, regulators). Moreover, the effective use of real-world evidence generation during the pandemic led both policymaker/advisors and industry representatives to believe an increased use of real-world evidence in routine practise would be favourable, however participants also recommended greater alignment on how to evaluate it in their assessment processes. A vaccine monitoring platform and continuous dialogue through the European Centre for Disease Prevention and Control (ECDC) and reliance on a joint advisory board comprising stakeholders from various organizations were recommended to ensure this harmonization.

### Conditional Marketing Authorisation (CMA)

#### Reported Strengths and Limitations of Pandemic Practices

CMA is a tool that was already available prior to the pandemic in routine practice for medicines addressing an unmet medical need, allowing authorisation earlier in the development cycle based on an immature dataset, under the condition that additional data will be submitted and reassessed annually by the CHMP [16]. While many considered the tool essential to ensure rapid access during a public health emergency, some participants noted that its expanded use led to a considerable accumulation of workload for both industry and regulators. This additional burden, while not a structural inefficiency of CMA itself, was seen as a consequence of its broader application under crisis conditions. Furthermore, several participants believed that limited communication and transparency around the process and its evolving requirements may have contributed to public hesitancy and misunderstanding.

#### Optimisation Avenues

Regarding the CMA, both groups agreed that it is a needed and established tool in routine practice. While CMA is already an effective tool, participants did point out actions that could optimise the use, based on pandemic learnings. Both policymakers/advisors and industry representatives highlighted the need for stricter eligibility requirements, i.e. that CMA should only be applied in the case of unmet medical need, and further, when CMA is used, it should be communicated better to the public to generate more trust and limit hesitancy. CMA is essential for routine and emergency practices but poses challenges due to the post-approval burden of assessors of additional evidence. There were differing views on creating a separate emergency pathway, with some suggesting an alternative emergency use authorization. Overall, industry representatives recommended maintaining or creating a new emergency pathway, while policymakers noted CMA’s value despite the increased workload it generates.

### Joint Procurement

#### Reported Strengths and Limitations of Pandemic Practices

A frequently mentioned measure during the COVID-19 pandemic was joint procurement. Participants reported that this centralized process accelerated market access and explain that this enabled new products to enter the entire European market simultaneously at a uniform price, improving equity in access to innovative medicines and avoiding parallel trading.

Most participants believed that joint procurement increased efficiency by centralizing negotiations to one European negotiation party instead of each member state negotiating separately. However, there were also limitations mentioned. Some participants reported criticism from member states regarding the negotiated prices, which were perceived too low. One policymaker/advisor explained that “*The European Commission is always in the dilemma that they have to take care of the public health of the community*,* of the population*,* but also they want to have a competitive pharma industry. While health ministers on the national level almost only have the perspective of purchasing goods for the public*,* and they don’t look whether the pharma companies are making enough money and whether they are competitive. So this double perspective of the European Commission is definitely difficult.”* (PMA8). Furthermore, various participants highlighted that some member states still negotiated their own prices with companies to secure better deals, leading to inefficient use of resources.

#### Optimisation Avenues

Some industry representatives expressed their desire to move towards a European system with one price for all member states (with joint clinical assessment as the first step). Further, one policymaker/advisor believed that joint procurement is a good tool to drive innovation in areas where there is a clear need. Overall there was no clear consensus within industry, nor policymakers/advisors, regarding the broader applicability outside of emergency context.

*“I think it would be beneficial for Europe to take this into their own hands and say for new drugs we start as of today and you put benchmarks. Then we have the prices of all the products in all the countries in Europe. In most of the countries the prices are publicly available. It’s possible to put benchmarks and say okay*,* you have a new drug*,* is it as good as something that exists? Okay*,* then you have to stay within the threshold of 90 to 105% for example.” (IND5)*.

*“National politicians want to do their own thing*,* they want to make their own decisions. They don’t want the European Commission to interfere with their national decisions whether to purchase something or not. So I do know that the European Commission wanted to purchase some drugs as well*,* like [name medicinal product]*,* but the member states were very much against it that the European Commission does this a second time.” (PMA8)*.

Some policymakers/advisors recommended that this action should be used in future health emergencies, but some raised concerns. A first proposed consideration related to the differences in healthcare systems across members states, and recommendation to differentiate prices in different countries depending on gross domestic product. Secondly, several policymakers/advisors pointed out to make sure that when member states sign-in on the joint procurement they would refrain from individually negotiating other contracts with companies for a better price. While most industry participants were cautious about applying joint procurement broadly in routine settings, some representatives highlighted potential benefits under specific conditions. These included avoiding parallel trade, supporting equitable access across Europe, reducing redundant data requests from member states, and streamlining joint clinical assessments. However, there was also consensus that pricing and reimbursement decisions should remain at the national level. These perspectives suggest that selective, well-defined applications of joint procurement mechanisms could be positively received by industry under the right circumstances.

### Stakeholder Perceptions on Future Optimisation Avenues

Considering the benefits and limitations of these pandemic actions, stakeholders provided recommendations on how these actions could be best implemented going forward for future health emergencies as well as in routine practice. Table 2 provides an overview of the participants’ perceptions of their application potential.

**Table 2:** Overview of participants’ perceptions toward the application of the measures during future health emergencies and routine practices. CT: clinical trial, EMA: European Medicines Agency

Pandemic measures	Application potential			
	During future health emergencies		In routine practice	
	Policymaker/ advisor	Industry representatives	Policymaker/ advisor	Industry representatives
Emergency Task Force	ü	ü	NA	NA
Rapid Scientific Advice	#	ü	û	ü
EMA Rolling Review	ü	ü	#	ü
Conditional Marketing Authorisation	#	ü	#	#
Joint Procurement	#	ü	#	#

Note. ü = high application potential, # = diverging responses within stakeholder group, û = low application potential, NA = not applicable

## Discussion

Results of this study show that perspectives of industry participants and policymakers/advisors often differed. For instance, industry participants hope that several facilitating measures or procedures (e.g. rolling review, rapid scientific advice) would be implemented in routine practice and certainly to be applied again in future health emergencies. They propose for those measures to be applied to products that address unmet medical needs. Similarly to the results presented in this study, a survey with 18 companies reported that participants believe that the rolling review could hold opportunities in the future for innovative medicines [17]. This is also in line with EMA’s COVID-19 lessons learned report (2023), which states that high-priority medicinal products that address unmet medical need should be prioritised for inclusion in rolling review processes and receive early scientific advice, outside of pandemics [3]. Further, it is believed that these high-priority products should be considered based on clear inclusion criteria to make efficient use of available resources [3]. In this study, policymakers/advisors pointed out the highly resource-intensive nature of these practices and found them unsustainable to be applied again if no additional resources are provided in future health emergencies. As for the rolling review, decreasing resource intensiveness could include requiring applicants to submit more mature, quality-assured data during each rolling review cycle, to reduce the number of review iterations and avoid repetitive clarification cycles [10]. It must be noted that benefits and limitations reported in Fig. 1 are causally linked to each other and cannot be controlled independently.

Recommendations resulting from reported pandemic experiences can be grouped in (i) procedural changes, (ii) dynamic allocation of recourses, (iii) clear inclusion criteria, and (iv) increased interaction and alignment between stakeholders and member states.

### Procedural changes to allow for more pragmatic regulatory and market access practices and reduction of administrative burden.

The findings of this study indicate that participants valued the pragmatic, hands-on approaches employed during the pandemic (i.e. rolling reviews, rapid scientific advice, CMA, and joint procurement). Firstly, results show that stakeholders believe that the submission requirements for the early scientific advice and EMA marketing authorisation could be reduced in length and extent without impacting the quality of assessment. The latter could for example be achieved under the form of cloud-based platforms to streamline real-time data submission, cross-regulator access, and evidence review [10], or artificial intelligence, which could accelerate information retrieval and synthesis or facilitate quantitative structure-activity relationships [18]. Moreover, the European Federation of Pharmaceutical Industries Association (EFPIA) proposes to apply a dynamic regulatory assessment to those products that address life-threatening diseases at first and in time to more medicinal products [19]. Secondly, the iterative nature of processes, best known from their application during the rolling review process, is believed by industry stakeholders to hold benefits for the routine practice [10, 17, 19].

### Dynamic Allocation of Resources According to Need During and Between Health Emergencies

While the primary determinant in reducing disease burden remains the development and availability of effective and safe medicines, accelerated regulatory processes and rapid scientific advice can still play a supportive role during health emergencies by enabling faster deployment and access once such products become available. In this context, additional efforts and resources for the EMA and network of assessors may still yield meaningful population-level benefits. Knowing that the productivity losses caused by the pandemic reached 2.1% of the gross domestic product [20], the associated regulatory costs could be considered minor compared to the broader potential gains in health outcomes and crisis preparedness, ultimately lowering costs for national health systems. Therefore, there is a need for a dynamic system where resources, both monetary and human, can be swiftly adjusted in times of pandemic need across the entire network. To address the discussed resource challenges, the EMA’s lessons learned report highlights the establishment of a joint EMA-Heads of Medicines Agencies tactical group focused on reinforcing the network’s capacity, a crucial aspect of crisis management [3].

### Clear Inclusion Criteria and Selection Process for Facilitating/Expediting Measures

To address capacity issues going forward, participants argued for more transparent and clear inclusion criteria for both rolling review and CMA. While it is difficult to predict in advance which products will successfully reach the market, the introduction of clearer prioritization criteria for regulatory support could help focus resources on those candidates most likely to address significant unmet medical needs. Such an approach may reduce the risk of overextending limited resources on lower-priority products during emergency situations. Although several inclusion and exclusion criteria currently exist for facilitating/expediting measures at both European and national levels, these criteria (including the unmet medical need concept) are often considered too broad or open to interpretation [21]. Similarly to the current study, another qualitative study emphasised the added value of the continued use of the rolling review, but with more selective inclusion criterion to optimise the use of EMA and member state resources, specifically, varying the timeline of the rolling review process based on the type of product and avoiding unnecessary premature submissions [3]. Furthermore, in relation to the PRIME scheme, suggestions have been made to clarify the inclusion criteria [22]. The EMA has responded by developing a decision tree to delineate eligibility [22]. This approach could be extended to other regulatory and market access measures, such as CMA and joint procurement.

The proposal for the revision of the EU pharmaceutical legislation introduces the concept of unmet medical need as a key eligibility criterion, where only medicinal products addressing such needs qualify for facilitating or expediting measures [23]. Nonetheless, many stakeholders do not agree with the definition and criteria currently put forward and request more dialogue and reflection [24, 25]. Given the high value placed on predictability by industry stakeholders, enhancing the clarity and application of this criterion should be a priority in future health policy.

Moreover, while several industry participants in our study recognized potential advantages of joint procurement beyond pandemic contexts, particularly in addressing medicines targeting high unmet medical need, under specific conditions, this more positive outlook contrasts with official industry position papers [26, 27]. These typically advocate for joint procurement to be reserved for exceptional cross-border health threats, citing concerns about complexity, market sustainability, and national competencies. This stands in contrast to academic perspectives, which have framed joint procurement as a foundational instrument for advancing a stronger and more integrated European Health Union [28]. For instance, researchers have argued that the experience of joint procurement during the COVID-19 pandemic reveals its potential to support long-term EU-level coordination in health security and pharmaceutical access [28].

### Increased Interaction and Alignment Between and Within Stakeholder Groups

Increased interaction was favoured in regulatory authorization, scientific advice, market access processes, and better communication to the public to generate trust in accelerated processes. The study reports increased interaction between the EMA and stakeholders during multiple steps of the authorization and scientific advice process, which was positively received. Cavaleri et al. stress the importance of sustained dialogue between the EMA’s ETF and the industry [3]. Greater alignment of regulatory flexibilities regionally and nationally can support better access to medicines [17]. There is a need to maintain efforts for global alignment on regulatory requirements and strengthen international cooperation beyond crisis situations [3]. International collaboration has been pivotal for harmonizing clinical trial requirements, supported by the ICMRA, but further strengthening is recommended [29]. EFPIA’s proposed dynamic regulatory assessment similarly also requires collaboration between different EU jurisdictions, dynamic information sharing, and expedited regulatory pathways [19].

Positive experiences were reported with increased interaction and alignment of marketing authorization practices with regulators outside the EU via the EMA’s OPEN initiative during the pandemic [30]. Participants recommend integrating this initiative into daily practice for easier tracking of marketing authorization assessments. Joint procurement of medicines at the EU level was positively received, though some stakeholders found it slow, as member states often prioritize their sovereignty over EU solidarity, posing challenges to joint procurement implementation [31].

An EU Parliament statement highlighted the need for more transparency in joint procurement contract negotiations [32]. Similarly to what was expressed by some participants in this study, EFPIA also reports that the European Commission would benefit from involving regulatory and scientific experts in the negotiating team [33]. Potential solutions include relying on expert knowledge, previous procurement experiences, and involving external partners and stakeholders [34].

During the COVID-19 pandemic, hesitancy was observed from both the public and governments in trusting the vaccines and antivirals [35–37]. To prevent hesitancy in future health emergencies, it is crucial to build and maintain trust while accelerating approval processes. Recent literature confirms the importance of transparent communication and real-time data sharing in maintaining public trust [38]. During this pandemic, EMA’s accelerated publication of safety data during COVID‑19 already aimed to enhance transparency [39]. Moreover, public and/or patient involvement in regulator and market access decision-making processes is expected to enhance trust further [40].

### Limitations and Strengths

This qualitative research builds on existing literature by combining perspectives from both policymakers/advisors and industry representatives on pandemic actions. The study’s strength lies in capturing both perspectives through a systematic approach, with data saturation of interviews methodologically assessed and achieved, and analysis performed by two researchers.

However, there are limitations inherent to qualitative research. The study is based on a purposive sample, so the conclusions are not meant to represent all stakeholders’ beliefs. Moreover, although snowball sampling facilitated efficient access to highly qualified and relevant experts, the authors acknowledge that this approach might limit the diversity of perspectives. To mitigate potential biases, initial contacts were deliberately selected across various stakeholder groups and geographical regions. Researchers aimed to include various profiles to gain diverse perspectives from regulatory and HTA policymakers/advisors and industry representatives. The study only included experts from large pharmaceutical companies and trade organizations, suggesting the need for future exploration of small and medium-sized enterprises’ perspectives. Additionally, there was a dominant representation of participants from Western European countries.

Lastly, the study focused on the experiences and perspectives of stakeholders directly involved in the COVID-19 response, and the results are limited to the challenges and optimization avenues they identified. Aspects of the Joint Procurement process such as supply criteria beyond price, safety and liability arrangements, the role of NITAG recommendations, allocation mechanisms, and funding structures were not raised by participants and are therefore not addressed in detail in the analysis. As a result, the findings may not capture the full complexity of the Joint Procurement process as implemented at the European level.

## Conclusion

This study provides an overview of stakeholder perceptions on regulatory and market access practices during COVID-19, highlighting how these experiences can inform regulatory and market access practices both in routine operations and during health emergencies. The most significant achievement during the COVID-19 pandemic was the increased speed at which new vaccines and therapeutics were made available to patients. However, this rapid pace also revealed challenges related to inefficiencies and limited transparency. To optimize the framework, there is a need for enhanced interaction and alignment among stakeholders, the development of more pragmatic procedures, and the application of clear and transparent inclusion criteria. These reflections are informative for improving our preparedness and responsiveness in future health crises.

## Supplementary Information

Below is the link to the electronic supplementary material.

Supplementary Material 1

Supplementary Material 2

Supplementary Material 3

Supplementary Material 4

## Data Availability

No datasets were generated or analysed during the current study.
