# Supplementary material for: Future-Proofing European Pharmaceutical Regulatory and Market Access Practices Based on EU Learnings from the COVID-19 Pandemic: Insights from Multi-Stakeholder Interviews
Source: Ther Innov Regul Sci. 2025 Sep 6;60(1):105–16. doi: 10.1007/s43441-025-00855-2 (PMC12753532; doi:10.1007/s43441-025-00855-2)
Supplement: Supplementary file 2 — Supplementary Material 2 [file 43441_2025_855_MOESM2_ESM.pdf]

Article title: Future-Proofing European Pharmaceutical Regulatory and Market Access Practices Based on Learnings from the COVID-19 Pandemic in the EU: Insights from Multi-Stakeholder Interviews

Authors' information: Zilke Claessens[1*], Grace Beirne[2], Catherine Decouttere[2], Nico Vandaele[2], Liese Barbier[1], **Isabelle Huys[1*]**

[1] Clinical Pharmacology and Pharmacotherapy, Department of Pharmaceutical and Pharmacological Sciences, KU Leuven, Leuven, Belgium
[2] Access-To-Medicines Research Centre, Faculty of Economics & Business, KU Leuven, Leuven, Belgium

*corresponding author: contact.Isabellehuys@kuleuven.be, Zilke.claessens@kuleuven.be

Supplementary file 2: Recommendations for optimisation

Legend: Policymakers/advisors | Industry | Both

Phase in Drug Life Cycle	Procedure	Recommendations			
		Provide additional monetary resources and personnel	Increased communication and transparency	Define clear eligibility criteria	Procedural changes
**Routine practice**					
**Regulatory scientific support**	**Scientific advice**	✓		✓	Implement the rapid review tool in routine practice<br>More pragmatic application procedure to facilitate and accelerate submissions
**Regulatory authorisation**	**Standard authorisation**				Simplified submission documents<br>More interactive, dynamic assessment process<br>One harmonized European assessment, applying to all member states
	**Rolling review**	✓		✓	Rolling review as an additional tool or replacement for PRIME and AA
	**Conditional marketing authorisation**		✓	✓	
**Market access**	**Joint procurement**			✓	Move towards a European system with one price for all member states (joint clinical assessment as the first step)

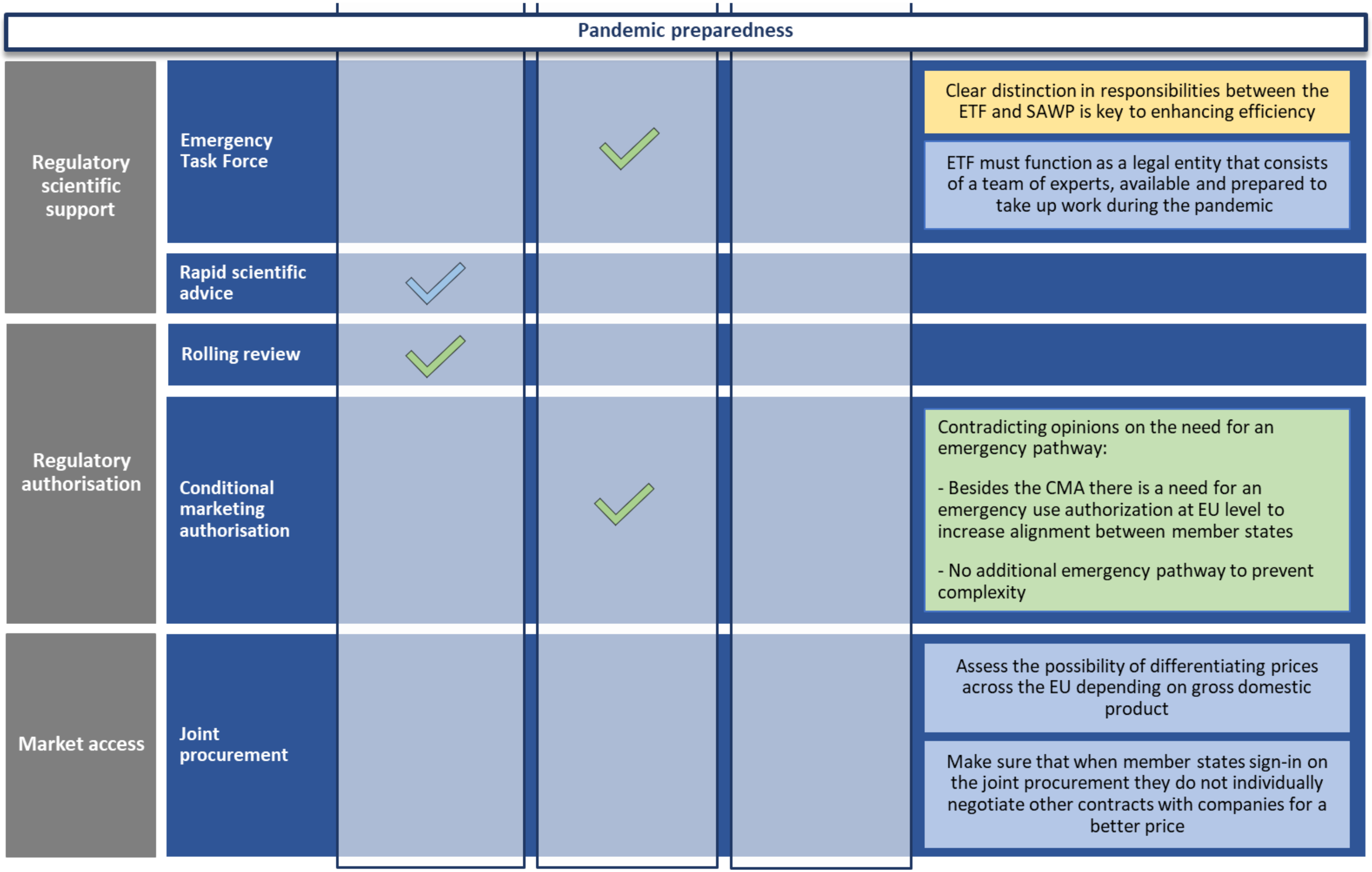

Figure 1. Recommendations for the optimisation of both routine and health emergency practice. AA: Accelerated Assessment, CMA: Conditional Marketing Authorisation, ETF: Emergency Taskforce, PRIME: Priority Medicines Scheme
