# Supplementary material for: Future-Proofing European Pharmaceutical Regulatory and Market Access Practices Based on EU Learnings from the COVID-19 Pandemic: Insights from Multi-Stakeholder Interviews
Source: Ther Innov Regul Sci. 2025 Sep 6;60(1):105–16. doi: 10.1007/s43441-025-00855-2 (PMC12753532; doi:10.1007/s43441-025-00855-2)
Supplement: Supplementary file 3 — Supplementary Material 3 [file 43441_2025_855_MOESM3_ESM.pdf]

Interview guide

Semi-structured interview

Introduction

- Present yourself

  First, allow me to present myself. My name is [name researcher] and I am a PhD researcher at the KU Leuven conducting research at [research unit] under the supervision of Professor [name supervising professor].
- Thank the interviewee for his/her participation in advance

  I would like to thank you for taking the time to participate in this interview. Your views, opinions, and experiences are very important to our research.
- Explain shortly the purpose of the interview:

  With this interview, we want to explore your insights and **views on the functioning of regulatory and market access processes** during the COVID-19 pandemic, the **challenges** in this context, and what you would identify as **possible optimisation avenues**. Interviews are organised not only with regulators, but also with HTA bodies, payers and industry experts. This study forms part of a PhD research and aims to inform a dynamic model of the drug development process from end to end, the downstream impact of these regulatory and market-access decisions on the health system and on patient outcomes.

  The interview will take about 1 hour.
- Put the interviewee at ease:
  - I want to emphasize that there are **no right or wrong answers** and that it is no problem if you might not know the answer. In that case, it would be great if you could recommend us the name of a contact person who could clarify this aspect.
  - This interview will be **digitally audio-recorded.** This makes it easier for us to process all information that is provided in the interviews. Anything you say today will be **completely confidential** and will be processed pseudonymously. This implies that we will not use your name or any identifying information in reports or publications following this study. The data collected today will be stored securely and viewed only by the researchers involved in this project. Participation is completely voluntary, you can withdraw at any point, without having to give an explanation. You do not have to answer any questions during the interview if you do not feel comfortable answering them.
- Do you have any questions before we start the interview?
- So, we will now start the recording. Is that OK?

Questions

The interview is conducted in a semi-structured fashion. The questions and topics vary depending on the expertise of the participant, so participants may be asked a subset of questions from this list, and/or potentially additional similar questions that are informed by the answers of the interviewee.

1. **Introductory questions**
   - Could you please briefly introduce yourself?
   - Can you tell us a bit more about your current position?
   - How did you experience these challenging times?

2. **Regulatory and market access measures**

- How would you describe the regulatory/market access flexibilities that were installed during the pandemic?
    - On which levels (procedures, pathways, practices) are regulatory flexibilities installed during the pandemic?
    - What are the challenges related to these increased flexibilities?
- Several regulatory pathways are in place to expedite authorization, can you explain which were crucial during the pandemic for COVID-19 products?

For every specific mentioned flexibility or measure following questions were asked:

- What is your perspective towards [the respective pandemic measure]?
- What are, in your opinion, the **advantages** of this measure?
- What are, if any, the **challenges** with this measure?
    - From regulatory point of view
    - From developers point of view
    - From HTA/payer point of view
        - What, if any, challenges do you see downstream in the drug life cycle – HTA, reimbursement, price setting?
- What are, if any, important opportunities or possibilities that this pathway offers?
- How do you see this pathway evolving over time?
- Could these specific emergency practices be extended to certain cases in **day-to-day practice**?
- What other alternative procedures/actions come to mind that have been used during this pandemic to speed up authorisation?

To summarize:

- Is the regulatory framework fit for the purpose of authorizing products during a global health emergency?
    - If not, where is it failing?
    - Which recommendations would you make to future-proof emergency practices?
- What are the most important learnings you've experienced during those emergency times, in regard to regulatory science?
- What do you think will be the long-lasting effects of this global health crisis on the regulatory framework and its functioning if there are?
- Can you list some recommendations you would like to make for the future of the regulatory framework?

3. Round-up questions

This brings us toward the final part of the interview. I have some final questions that I would like to ask

- *Do you want to add anything else to the interview?*
- *Do you want to emphasize something of what we discussed? What would be for you the key takeaway from the interview?*
- *Do you think we forgot something in what we discussed?*
- *Do you have any questions for me?*
- *Do you have any suggestions to take into account for our research?*
- *Do you have a suggestion for other interesting interviewees concerning this topic?*
