# Supplementary material for: Future-Proofing European Pharmaceutical Regulatory and Market Access Practices Based on EU Learnings from the COVID-19 Pandemic: Insights from Multi-Stakeholder Interviews
Source: Ther Innov Regul Sci. 2025 Sep 6;60(1):105–16. doi: 10.1007/s43441-025-00855-2 (PMC12753532; doi:10.1007/s43441-025-00855-2)
Supplement: Supplementary file 4 — Supplementary Material 4 [file 43441_2025_855_MOESM4_ESM.pdf]

Article title: Future-Proofing European Pharmaceutical Regulatory and Market Access Practices Based on Learnings from the COVID-19 Pandemic in the EU: Insights from Multi-Stakeholder Interviews

Authors' information: Zilke Claessens[1*], Grace Beirne[2], Catherine Decouttere[2], Nico Vandaele[2], Liese Barbier[1], **Isabelle Huys[1*]**

[1] Clinical Pharmacology and Pharmacotherapy, Department of Pharmaceutical and Pharmacological Sciences, KU Leuven, Leuven, Belgium

[2] Access-To-Medicines Research Centre, Faculty of Economics & Business, KU Leuven, Leuven, Belgium

*corresponding author: contact.Isabellehuys@kuleuven.be, Zilke.claessens@kuleuven.be

Supplementary file 4: Map of pandemic measures identified during interviews

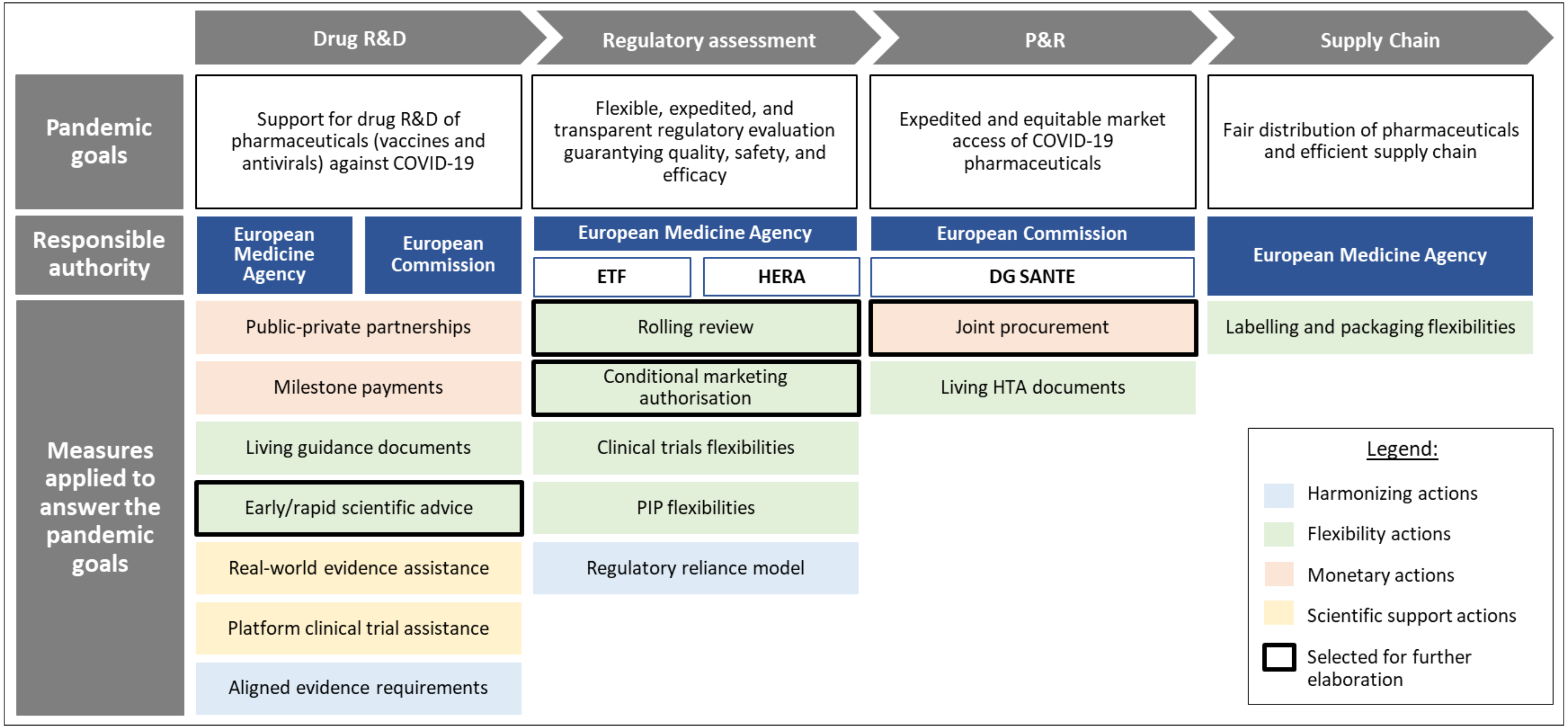

Figure 1. Overview of pandemic goals and respective flexibility measures applied to reach these goals. *ETF: Emergency task force, HERA: Health Emergency Preparedness and Response Authority, R&D: research and development, P&R: pricing and reimbursement, PIP: Paediatric Investigation Plan.*
